# Using Emergency Department Data to Inform Specialty Strategy: Analyzing the Distribution of 13,777 Consecutive Immediate Orthopaedic Consults in an Urban Community Emergency Department

**DOI:** 10.5435/JAAOSGlobal-D-20-00005

**Published:** 2020-02-12

**Authors:** Ariel R. C. Silverman, Jalen N. Broome, Richard C. Jarvis, Grace C. Plassche, Ira H. Kirschenbaum

**Affiliations:** From the Department of Orthopaedics (Ms. Silverman and Dr. Kirschenbaum), BronxCare Health System, The Bronx, NY, the Anne Arundel Orthopaedic Surgeons, Annapolis, MD (Mr. Broome), and the Emory School of Medicine, Atlanta, GA (Mr. Jarvis); and the Orthopaedic Foundation for Active Lifestyles (Ms. Plassche).

## Abstract

Although an estimated 20% of emergency department (ED) visits are musculoskeletal in nature, it is unclear which of these require urgent orthopaedic consultation and which orthopaedic subspecialties are best suited for these consults. When an ED's internal staff does not have necessary orthopaedic coverage, transfer to other facilities is required. However, transfers pose major inconveniences and financial burdens to patients and can lead to long ED wait times, hospital financial loss from walk outs, decreased patient safety, and decreased staff morale. Therefore, it is beneficial for a hospital to have the appropriate staff readily available for consults. Data can be used to assess the orthopaedic needs of an ED. We evaluated the nature of urgent ED consults requesting the timely presence of an orthopaedic provider.

**Methods:**

Between the years 2008 and 2017, the Orthopaedics Department of this Health System saw 13,777 patients from the ED requesting immediate consult from an orthopaedic provider. We retrospectively analyzed the distribution of anatomic areas and nature of these injuries for these visits.

**Results:**

Hand, foot, and ankle consults comprised 75% of the volume. Knee, hip, and spine accounted for 15% of consults. Most injuries were fractures. Infections and sprains were also common.

**Discussion:**

By determining and understanding this distribution, orthopaedic departments can improve their organization to better respond to urgent ED consults, allowing for the proper delivery of orthopaedic point-of-care service to patients, increased revenue for the hospital, proper availability of core competencies, and increased value to the healthcare delivery system as a whole. We also believe that the trends observed in our data are largely generalizable to EDs serving urban communities similar to ours. Thus, these results can help inform a synergistic strategy for the system comprising EDs, urgent care clinics, and orthopaedic surgeons servicing them.

The emergency department (ED) of any hospital treats and subsequently either admits or discharges a large number of patients over the course of a given year. Although emergency physicians represent less than 5% of doctors, they treat a quarter of all acute care encounters and more than half of such visits by the uninsured such that they provide more acute care to Medicaid beneficiaries and the uninsured than the rest of the physicians in the United States combined.^1,2^ This is particularly important in urban populations such as that of our Health System, in which the ED treated over 133,000 patients in 2018, approximately 74% of which were adults. The designated ED physicians treat most of these patients, but they refer for immediate consultation for patients with specific conditions that would benefit from being escalated to a specialty clinical service.

It has been estimated that approximately 20% of all ED visits are musculoskeletal in origin.^3^ Although most patients are treated and discharged by the ED physician, many do require the immediate and/or timely in-person consultation and subsequent intervention by an orthopaedic surgeon/provider. There are important issues surrounding these consultations including availability of an orthopaedic surgeon in a timely manner, compensation of that physician for his or her services, the core competencies of the consulting orthopaedic surgeon in treating a wide range of musculoskeletal conditions, and the epidemiology of the specific conditions that orthopaedic surgeons are requested to consult on urgently. A survey by the American College of Emergency Physicians showed that 66% of EDs had insufficient subspecialty coverage and that this was coupled with an increase in the number of transfers from one ED to another for this care.^4^ It is unclear whether the lack of availability of subspecialty care was because of unwillingness of the available providers to provide consults or insufficient availability of the specialty providers on staff owing to poor estimation in predicting the types of consults the ED would require responses to and recruiting the appropriate physicians accordingly. In any event, when problems with these cases arise, it usually results in transfer to another ED.

It is reasonable to assume that EDs, by design, develop relationships with area hospitals that support the necessary specialty coverage. However, such transfers can pose major inconveniences to patients and their families, as well as major financial burdens that may be altogether unnecessary.^4^ In addition to decreased patient satisfaction, long ED wait times result in financial loss from walk outs, decreased patient safety, and decreased staff morale.^5^ Based on this crisis in specialty care–related ED visits, we decided to evaluate the nature of urgent consults requesting the timely presence of an orthopaedic provider in our busy, urban ED.

Although this Health System is not a certified trauma center, its emergency department cares for a community in the South Bronx with an approximate catchment area of over 1.4 million people, and currently delivers over 130,000 visits per year. Between the years 2008 and 2017, the Orthopaedics Department of this Health System saw over 28,000 ED and inpatient consults. 13,777 of these patients were consults from the ED requesting immediate presence of an orthopaedic provider.

As noted above, although an estimated 20% of visits are musculoskeletal in most EDs, it is unclear which of these require urgent orthopaedic consultation and in addition which orthopaedic subspecialties are best suited for these consults. Therefore, information is insufficient for an orthopaedic department to recruit staff appropriately to meet this need. As a model to illustrate how we believe an orthopaedic department can use data to properly equip itself with the core competencies to meet the patient needs of the community that the ED serves, we completed an analysis on the aforementioned 13,777 visits. We believe that by determining and understanding this distribution, orthopaedic departments can improve their constructions to be better equipped to respond to urgent ED consults, allowing for the proper delivery of orthopaedic point-of-care service to the patients, increased revenue for the hospital, and increased value to the healthcare delivery system as a whole. We also believe that the trends observed in our data will be largely generalizable to EDs serving urban communities such as ours. Thus, this could help inform a better synergistic strategy for the system comprised EDs, urgent care clinics, and orthopaedic departments.

## Method

We performed a retrospective analysis of orthopaedic consults from the ED between April 21, 2008, and June 25, 2017, using the department's care management data platform. This is a custom database written on an open-source, low-code database platform (TrackVia). This database was programmed by the senior author. Information from the electronic medical record was transferred to this care management platform to facilitate rounding, daily care management, sign outs, discharge planning, and recording of complications. In addition to managing the daily consults from the ED and the hospital floors, the platform managed the surgical episode of care from surgical indications to discharge and long-term follow-up.^6^ Although initially implemented for improved care management, this platform has incidentally allowed for research and business intelligence through the development of categorical capabilities to drill down on the information needed to develop care and management strategies. On TrackVia, primary orthopaedic providers (physician assistants, fellows, or attendings) are required to enter the consult, its source, and a variety of information concerning diagnosis and treatment. Through this platform, we were able to isolate and analyze 13,777 consecutive consults that originated in the ED and then subsequently requested immediate orthopaedic consult, which was fulfilled by one of the providers of the orthopaedic department. These patient cases were then documented in TrackVia, which allowed us to analyze the distribution of anatomic locations and additional presence and patterns of fracture location, which played a role in dictating which of the orthopaedic subspecialties was best suited to treat the patient.

## Results

**Figure 1:**
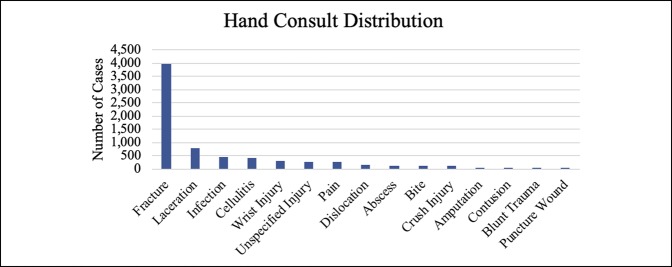
Chart illustrating the hand consult type ranked by incidence.

**Figure 2:**
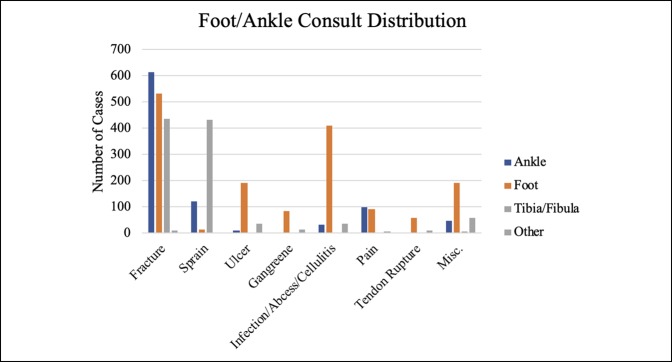
Chart illustrating the foot/ankle consult distribution by anatomical area.

**Figure 3:**
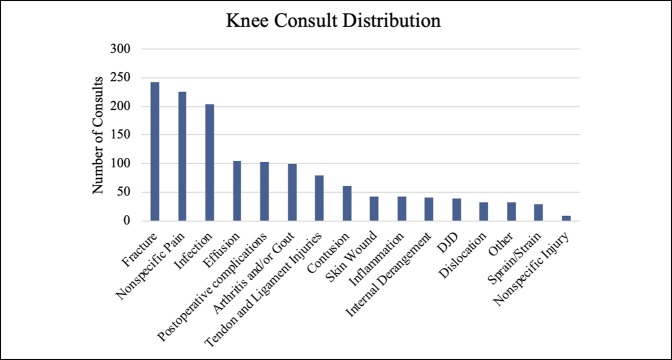
Chart illustrating the knee consult type ranked by incidence.

**Figure 4:**
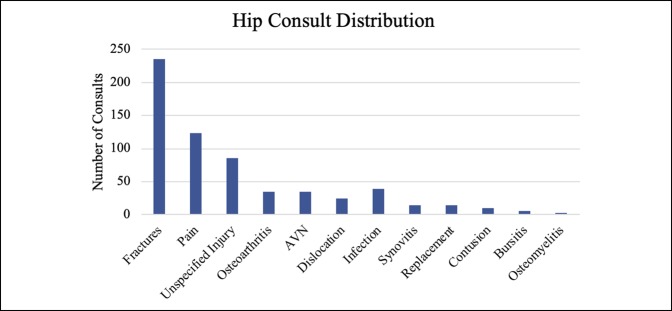
Chart illustrating the hip consult type ranked by incidence.

**Figure 5:**
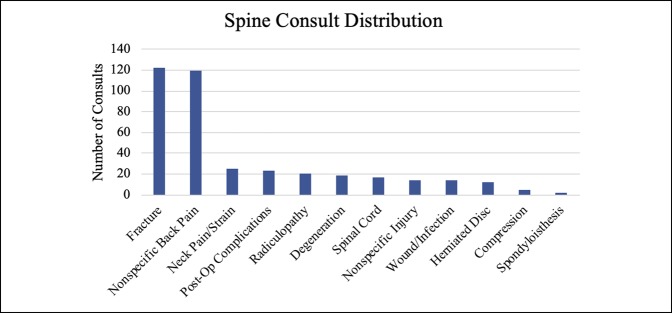
Chart illustrating the spine consult type ranked by incidence.

**Figure 6:**
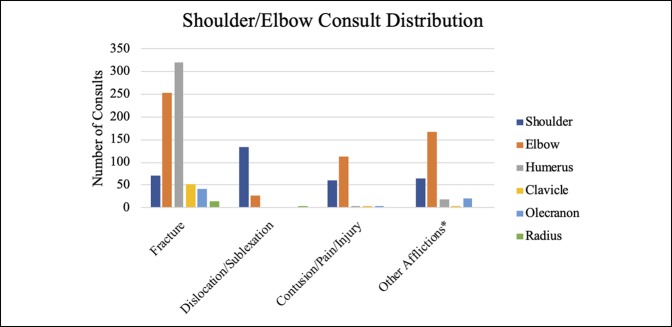
Chart illustrating the shoulder/elbow consult distribution by anatomical area.

**Figure 7:**
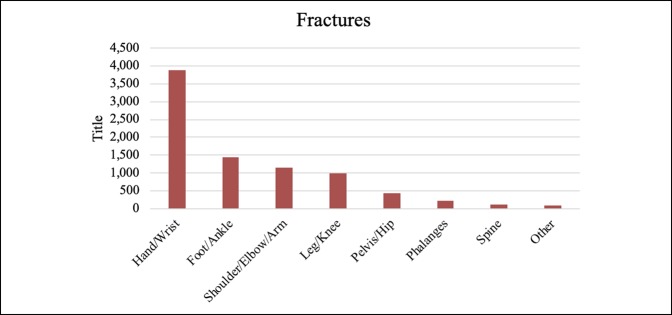
Chart illustrating the fracture incidence by area.

Together, hand, foot, and ankle consults comprised approximately 75% of the overall volume, with hand consults accounting for approximately 50% of the consults. Knee, hip, and spine were also significant contributors, although together they made up only 15% of the total volume (Table 1). Fractures, calculated separately, accounted for most (60.61%) of the visits. Figures 1–6 show the nature of the distribution of the type of consults in each anatomical area. In hand, fractures were approximately five times more prevalent than the nearest injuries, which were laceration, infection, and cellulitis (Figure 1). Foot and ankle consults were also dominated by fractures, as well as sprains and infection (Figure 2). The three most common consults related to the knee were fracture, nonspecific pain, and infection (Figure 3). Hip consults were dominated by fractures and nonspecific pain, which was similar to the spine consult distribution (Figures 4 and 5). Shoulder/elbow consults, too, were dominated by fractures and dislocations, with a significant number of contusions present (Figure 6). Fractures most commonly occurred in the hand/wrist, followed by foot/ankle, shoulder/elbow/arm, leg/knee, pelvis/hip, phalanges, spine, and then “other” (Figure 7).

**Table 1:** Table Shows the Distribution of Total ED Consults Based on the Location of Injury

Anatomical Area	No. of Visits	Percentage of Total Visits
Hand	7,036	51.07%
Foot/ankle	3,319	24.09%
Knee	1,158	8.41%
Hip	576	4.18%
Spine	353	2.56%
Shoulder/elbow	1,335	9.69%
Total ED consults	13,777	100%

ED = Emergency Department

## Discussion

As we enter an era of value-based medicine, large multispecialty private and hospital-based groups, and new types of reimbursement strategies based on outcomes and cost, gaining a better understanding of the nature of the referrals from EDs that require the presence of an orthopaedic surgeon or orthopaedic allied health professional (orthopaedic physician's assistant or orthopaedic nurse practitioner) is critical in constructing a proper orthopaedic emergency care delivery system.

This study was carried out in a safety net hospital, which treats individuals regardless of their insurance status. Thus, we have reason to believe that the distribution of orthopaedic ED consults represents the actual needs of the community, regardless of the socioeconomic status of individual patients. Our results show the distribution of orthopaedic ED consults by percentage over the course of 2008 to 2017. Most injuries occurred in the hand (51%), with foot/ankle (24%) following as the second most common subspecialty. Studies performed in other populations have also found hand injuries to be most prevalent in EDs and have also determined that hand and wrist injuries are the most expensive to treat, with productivity costs owing to missed work representing the most significant portion of the economic burden.^7^ However, in other populations, hand and wrist conditions, although still the most prevalent anatomical area being treated, accounted for only one of every 10 patients, with nearly half requiring surgery.^8^ Because this represents less than one-fifth of the frequency we found in our population, this emphasizes the importance of understanding the distribution within one's specific catchment area to properly care for the community. Regardless, there are certain trends that can be predicted and generalized. For example, this disproportionate frequency of hand/wrist injuries is to be logically expected of a large working population often involved in manual labor. In addition, the nonelderly patient population entering the ED is more likely to be treated for surgical (rather than medical) conditions and more likely to be admitted for injuries, such as fractures.^9^ It tends to be the case that in conditions related to the hand, trauma-related visits decrease with age and degenerative conditions increase with age.^8^ Insights such as these can help inform strategy, although we believe that a specific analysis of one's own patient population will lead to a significant improvement in the ability to properly prepare for volume according to the nature of the injury. Although it was previously known that we receive many hand and foot consults, this provides the quantitative evidence to support staffing multiple hand and foot surgeons such that the hospital can maximize its curative potential by meeting the needs of the ED and the community. This also confirms that our findings are in line with the distributions of both injury type and cost in other healthcare systems.

Specialty coverage of an ED has long been an important issue in orthopaedics. Despite Medicaid expansion, access to outpatient orthopaedic care for Medicaid patients remains significantly limited.^10,11^ An orthopaedic department must reliably generate its own elective and outpatient surgery business while also leaving additional capacity for the inevitable ED consults that will require treatment. Although it is impossible to predict exactly what type of consult will be required on a given day, using aggregated data revealing past trends will undoubtedly provide some helpful insight in developing predictive analytics that can help inform staffing strategy. From a management perspective, outsourcing represents a loss in potential revenue for the hospital. In addition, it is advantageous to find an effective balance so that certain staff is not overwhelmed while others remain far below capacity. From a patient care perspective, it is important that there be the appropriate orthopaedic specialists available to be on site to deliver care so that outsourcing, which poses a potential financial burden and annoyance to the patient, can be avoided.^4^ Patients of low socioeconomic status face barriers to accessing specialty surgical care. It has been shown that patients with Medicaid coverage or no insurance are less likely to be offered local surgical care and are less likely to have the personal resources to reach distant centers for nonemergency care.^11^ Thus, this makes it particularly important that the EDs these patients enter are able to provide proper care on the site because this may represent the patient's only option. In addition, it has been shown the patients sometimes leave without treatment when there is a long wait time or the out-of-pocket costs are expected to be high.^3^

It has also been shown that ED closure in urban areas is increasing, with safety-net status and low-profit margin representing factors for increased risk of ED closure.^2^ Other research has suggested that urgent care centers may be well equipped to handle some of the volume EDs face. It was estimated that 13% to 27% of all ED visits could be dealt with by urgent care centers saving around $4.4 billion annually.^3^ The same study estimated that for trauma-related diagnoses, such as strains or fractures, 25% to 50% could be treated at urgent care centers. This may not be accurate, though, because access to orthopaedic surgeons with core competencies in these treatments needs to be available to urgent care centers as well. The increase in size and quantity of specialty orthopaedic hospitals has also had the effect of draining general hospitals of orthopaedic surgeons who normally covered their ED.

Current discussions and controversies in the literature have centered on issues such as the requirement for on-call responsibilities, core competencies of the on-call physician, reimbursements for on-call physicians, increased reimbursement per case for after-hours call, the appropriateness of transfers from one hospital to another, and follow-up after orthopaedic ED visits, to name a few.^1,8,12,13^ Because fee-for-service and small group orthopaedic practice have given way to capitated agreements, bundled payments, various forms of value-based medical delivery, large integrated practice units, and increased employment of physicians, the solutions to the problems of access to emergent specialty care are being shifted to the physician and nonphysician administrators who will need to develop comprehensive programs to solve these problems.^8,10,12^

Physician leaders of orthopaedic departments have to make critical staffing decisions. Depending on the nature of the hospital and its role in a health system, staffing decisions can be quite different. In general terms, staffing decisions can incorporate either a supply-side or demand-side approach to the decision-making process. An example of supply-side decision-making is a tertiary care hospital dedicated to few specialties. Because this hospital does not generally have responsibilities to care for a full community or an obligation to act as a vertical delivery system, it can decide to “supply” whatever orthopaedic services it wishes to. Some of these hospitals may have limited EDs and may not be a “911”-receiving hospital. Hospitals that either service large communities or are integrated into a vertical delivery system must develop their department staffing to match the “demands” of the community. The results from this study are clear; if one is organizing an orthopaedic delivery system that needs to respond to the demands of an urban population, it is critical to assure that there are adequate hand surgeons and foot and ankle surgeons available because these subspecialties represent approximately 75% of all reasons why an orthopaedic provider would need to provide an immediate orthopaedic consult to the ED. In addition, another important downstream action from our data would be to develop education and training for the ED in hand/wrist and foot/ankle issues. Focusing on this would certainly diminish the need to defer to orthopaedic specialists for all coverage, especially for minor cases. In an era of even more increasing subspecialization, the importance of ensuring that orthopaedic surgeons are equipped with a certain set of core competencies is increasing.
